# Antiphospholipid antibodies are persistently positive at high titers. Additive value of platelet-bound C4d

**DOI:** 10.3389/fimmu.2022.949919

**Published:** 2022-08-10

**Authors:** Savino Sciascia, Rory Bloch, Tyler O’Malley, Anja Kammesheidt, Roberta Vezza Alexander

**Affiliations:** ^1^ University Center of Excellence on Nephrologic, Rheumatologic and Rare Diseases (ERK-net, ERN-Reconnect and RITA-ERN Member) with Nephrology and Dialysis Unit and Center of Immuno-Rheumatology and Rare Diseases (CMID), Coordinating Center of the Interregional Network for Rare Diseases of Piedmont and Aosta Valley (North-West Italy), San Giovanni Bosco Hub Hospital, and Department of Clinical and Biological Sciences of the University of Turin, Turin, Italy; ^2^ Exagen Inc., Vista, CA, United States

**Keywords:** antiphospholipid antibodies, antiphospholipid syndrome, biomarkers, persistency, cell-bound complement activation products, PC4d

## Abstract

**Background:**

Classification criteria for antiphospholipid syndrome (APS) require that antiphospholipid antibody (aPL) positivity is confirmed after at least 12 weeks. We tested the hypothesis that aPL at high titers remain positive while low titers fluctuate over time. As both platelet-bound C4d (PC4d) and aPL are associated with thrombosis in systemic lupus erythematosus (SLE), we also evaluated whether PC4d can aid in APS diagnosis.

**Methods:**

Data from serum or plasma sent to Exagen’s laboratory for routine aPL testing were analyzed. Anti-cardiolipin (aCL) and anti-beta2 glycoprotein-1 antibodies (aB2GP1) were measured by chemiluminescence or ELiA fluorescence enzyme immunoassay; anti-phosphatidylserine/prothrombin complex antibodies (aPS/PT) by ELISA; PC4d by flow cytometry. Statistical analysis included descriptive statistics, logistic regression, and Pearson correlation.

**Results:**

More than 80% of positive samples with aCL and aB2GP1 at high titers - but not low titers - were positive at a retest. Non-criteria aPL (aPS/PT) followed a similar trend. aCL and aB2GP1 measured with two different technologies were highly correlated. PC4d and IgG of the three aPL were at best moderately correlated even when only positive aPL samples were analyzed (coefficient: 0.1917 to 0.2649).

**Conclusions:**

High titers aPL are often persistently positive, allowing an earlier diagnosis and risk assessment at the time of the initial screening. Conversely, a retest may be necessary for low titers. The high correlation between two methodologies suggests that these findings are independent of assay platform. The low to moderate correlation between PC4d and aPL might suggest a possible additive value to evaluate association with thrombosis in autoimmune diseases.

## Introduction

Antiphospholipid antibodies (aPL) are autoantibodies directed against plasma proteins complexed with negatively charged phospholipids on cell membranes (1). They are commonly found in patients with systemic lupus erythematosus (SLE) (2, 3) and are the main autoantibodies in patients with the antiphospholipid syndrome (APS) (4).

Binding of aPL to their autoantigen results in activation of complement and of endothelial cells, platelets, neutrophils, and monocytes (4). Activation of these cells promotes inflammation, clot formation, vasculopathy, thrombosis, and pregnancy complications in patients with APS (4) and SLE (2).

The classification criteria for APS (5) include lupus anticoagulant (LAC), as well as the IgG and IgM isotypes of anti-cardiolipin (aCL) and anti-beta2-glycoprotein 1 (aB2GP1), while some classification criteria for SLE include also the IgA isotypes (6–8). aPL titers are taken into consideration in all criteria.

In addition to LAC, aCL, and aB2GP1, so-called non-criteria aPL are common in APS and SLE (9, 10). IgG and IgM against the phosphatidylserine/prothrombin complex (aPS/PT) are among the best characterized non-criteria aPL (11, 12) and may have clinical significance in increasing risk of thrombosis (13, 14).

Classification criteria for SLE require only one aPL determination (6–8), while criteria for APS (5) require a confirmatory assay performed at least 12 weeks later to establish persistent positivity. In fact, transiently positive aPL, especially at low titer, may be the consequence of infections or medications and may not be clinically relevant (4, 15). Although evaluation of persistent positivity is important, a wait period of 12 weeks may delay risk assessment and adequate patient treatment. Persistent positivity for aPL has been evaluated in patients enrolled in the APS ACTION Registry, which includes patients with or without systemic autoimmune diseases but persistently positive for APS criteria aPL. Approximately 80% of patients with clinically meaningful aPL positivity at baseline remained stable at a median follow-up of 5 years, suggesting that high titer aPL may remain positive over time (15).

To extend these findings, we addressed persistent positivity of aPL by analyzing a large number of samples that were received by Exagen’s clinical laboratory for routine testing over the course of 5 years. In addition to the three isotypes of aCL and aB2GP1, we also evaluated persistent positivity of aPS/PT.

Assays for aPL are not uniformly standardized (16, 17) and the use different antigens, methodologies, and detection systems may lead to different results. Thus, we evaluated the correlation between two assay platforms.

Complement activation is involved in the pathogenesis of APS and SLE, and data have shown that aPL can activate the complement cascade (18, 19). Cell-bound complement activation products (CB-CAPs) are complement split products bound to blood cells, including erythrocytes, B cells, and platelets (20, 21). We and others have shown association of platelet-bound C4d (PC4d) with thrombotic events in SLE (14, 22–24). Thus, the third objective of this study was to evaluate the correlation between aPL and PC4d to investigate whether they have additive value in the assessment of patients with autoimmune diseases.

## Materials and methods

### Anti-phospholipid antibodies

All samples analyzed for this study were collected in the United States. All assays were carried out in Exagen’s clinical laboratory following the manufacturers’ instructions and Exagen’s standard operating procedures. aCL and aB2GP1 were measured by chemiluminescence (QUANTA Flash; Werfen, San Diego, CA) or ELiA fluorescence enzyme immunoassay (Phadia; ThermoFisher Scientific, Freiburg, Germany) in serum or plasma from venous blood collected in ethylenediamine-tetraacetic acid (EDTA). aPS/PT were measured by ELISA (QUANTA Lite; Werfen) and were considered positive if > 30 units (U). All the isotypes of aCL and aB2GP1 measured by chemiluminescence were considered positive if > 20 chemiluminescent units (CU); aCL IgG and IgM measured by ELiA were considered positive if > 40 units/ml; aCL IgA measured by ELiA was considered positive if > 20 units/ml; the isotypes of aB2GP1 measured by ELiA were considered positive if > 10 units/ml. Exagen measures aCL and aB2GP1 isotypes IgG and IgM as a panel. Thus, these four biomarkers are measured for all - or most - samples from the same specimen. aCL IgA, aB2GP1 IgA, and aPS/PT IgG and IgM are not part of the panel and are performed separately.

### Platelet-bound C4d

PC4d was measured by flow cytometry following Exagen’s standard operating procedures, as described (14, 23). The assay was extensively validated by Exagen before it was made commercially available, and validation was approved by the New York State Department of Health. Briefly, red blood cells from EDTA-anticoagulated blood were lysed and platelets were stained using a mouse monoclonal antibody against human C4d (Quidel, San Diego, CA) or a mouse IgG1 isotype control (MPOC-21). After incubation, samples were stained with a goat anti-mouse antibody conjugated to fluorescein isothiocyanate (FITC). A mouse anti-human monoclonal antibody against human CD42b conjugated to phycoerythrin (PE) was used to identify platelets. FACS analysis was performed using a Gallios flow cytometer (Beckman Coulter, Brea, California). Light scatter (forward and side) gating parameters were used to isolate the platelet population, followed by secondary gating based on positive CD42b PE staining. Quantification of the non-specific (isotype control) and specific (C4d) fluorescence was determined for the CD42b PE gated platelets (5000 events). Net MFI was calculated by subtraction of isotype control MFI from the specific C4d MFI on gated platelets.

### Patient population

Data of samples sent to Exagen’s clinical laboratory for routine patient testing were extracted from Exagen database. Approximately 36,000 individuals who had at least two determinations for aCL IgG, aCL IgM, aB2GP1 IgG, and aB2GP1 IgM over a 5-year period (from November 2016 to October 2021) were identified. The number was lower (approximately 19,000) for the IgA isotypes and for aPS/PT. Only the samples for whom the first determination was positive were analyzed. If a patient was tested more than once after a positive determination, only the first subsequent determination was included in the analysis.

### Statistical analysis

Descriptive statistics were performed to evaluate persistent positivity of the biomarkers by quartile analysis, performed by dividing the number of observations into four equal parts. Logistic regression analysis was performed to evaluate covariates associated with persistent positivity. Pearson correlation analysis was performed to evaluate correlation between two platforms for aPL measurement, and between aPL and PC4d.

## Results

### aPL persistency

Of the approximately 36,000 or 19,000 individuals who had at least two aPL determinations, we analyzed only the samples for whom the first aPL determination was positive. The interval of time between the first and the second determination ranged between 0 and 252 weeks. The median time ranged from 54.07 weeks for aB2GP1 IgG (IQR = 24.0 to 95.14 weeks) to 61.00 weeks for aPS/PT IgG (IQR = 30.71 to 106.71 weeks) (**Supplementary Table 1**). The number of patients who were tested at least twice and for whom a certain aPL isotype was positive at the first determination is reported in **Table 1**.

**Table 1:** aPL positivity at retest.

		Positive samples at retest/total samples	Percent
aCL IgG N=2,964	Q1 (20-25 CU)	334/741	45.1%
	Q2 (25-32 CU)	514/741	69.4%
	Q3 (32-52 CU)	627/741	84.6%
	Q4 (52-2024 CU)	709/741	95.7%
aCL IgM N=1,653	Q1 (21-27 CU)	196/414	47.3%
	Q2 (27-38 CU)	294/413	71.2%
	Q3 (38-69 CU)	363/413	87.9%
	Q4 (69-774 CU)	398/413	96.4%
aCL IgA N=553	Q1 (21-26 CU)	47/139	33.8%
	Q2 (26-35 CU)	86/138	62.3%
	Q3 (35-64 CU)	122/138	88.4%
	Q4 (66-352 CU)	134/138	97.1%
aB2GP1 IgG N=1,664	Q1 (21-30 CU)	164/416	39.4%
	Q2 (30-57 CU)	283/416	68.0%
	Q3 (57-200 CU)	368/416	88.5%
	Q4 (201-6100 CU)	408/416	98.1%
aB2GP1 IgM N=948	Q1 (21-29 CU)	115/237	48.5%
	Q2 (29-45 CU)	186/237	78.5%
	Q3(45-88 CU)	209/237	88.2%
	Q4 (89-841 CU)	228/237	96.2%
aB2GP1 IgA N=368	Q1 (21-27 CU)	51/92	55.4%
	Q2 (27-40 CU)	82/92	89.1%
	Q3 (41-80 CU)	89/92	96.7%
	Q4 (80-512 CU)	91/92	98.9%
aPS/PT IgG N=1,073	Q1 (31-36 CU)	62/269	23.1%
	Q2 (36-45 U)	103/268	38.4%
	Q3 (46-70 (U)	162/268	60.5%
	Q4 (70-150 U)	242/268	90.3%
aPS/PT IgM N=3,217	Q1 (31-36 U)	285/805	35.4%
	Q2 (36-47 U)	390/804	48.5%
	Q3 (47-80 U)	555/804	69.0%
	Q4 (80-150 U)	718/804	89.3%

Samples positive for the specified isotype (IgG, IgM, and IgA) of anti-cardiolipin antibodies (aCL), anti-beta2 glycoprotein-1 antibodies (aB2GP1), or anti-phosphatidylserine/prothrombin complex antibodies (aPS/PT) and for which a second determination was available were divided in quartiles (Q) based on their titers. The range in each quartile is reported in parenthesis. N indicates the number of samples that were positive at the first test. Percent indicates the percent of positive samples that gave positive results at the first subsequent test in each quartile. All aCL and aB2GP1 isotypes were measured by chemiluminescence (QUANTA Flash; Werfen) and were considered positive if > 20 chemiluminescent units (CU); aPS/PT isotypes were measured by ELISA (QUANTA Lite; Werfen) and were considered positive if > 30 units (U).

Because our analysis was based on real word data and aimed to evaluate persistent positivity regardless of the interval of time required by the classification criteria for APS, all samples were included in the analysis, irrespective of the time between determinations. However, the majority of samples were retested after more than 12 weeks. Depending on the particular analyte, 92% to 95% of samples were tested more than 12 weeks apart. In addition, 98% to 99% of samples were tested more than 30 days apart. Positivity rate of samples tested for the IgG of aCL and aB2GP1 at least 12 weeks, 30 days, or 7 days apart is reported in **Supplementary Table 2.**

To evaluate aPL persistent positivity over time, we performed a quartile analysis for the isotypes of aCL and aB2GP1 measured by QUANTA Flash and the isotypes of aPS/PT measured by QUANTA Lite. Confirmation rates of each aPL show that, for all biomarkers, percent positivity at the retest increased as the titer of the first positive determination increased. Confirmation rate was the lowest in the first quartile and ranged from 23.1% for aPS/PT IgG to 55.4% for aB2GP1 IgA. On the other hand, confirmation rate was highest in the fourth quartile and ranged from 89.3% for aPS/PT IgM to 98.9% for aB2GP1 IgA (**Table 1**).

As expected based on literature data (25), a higher percentage of double positive samples (positive for the IgG of aCL and aB2GP1) were positive at retest compared to single positive samples. In fact, 94.4% of double positive samples tested positive at retest for one of these aPL, while 61.4% to 68.8% single positive samples tested positive at retest (**Supplementary Table 3**). Interestingly, no samples positive for both IgM aCL and IgM aB2GP1 were IgG negative.

Logistic regression analysis showed that the strongest predictor for retest positivity for all the aPL was the initial quantitative positive value of the same analyte, while positivity for a different aPL, gender, age, or time between visits were not associated with probability of testing positive at the retest (data not shown).

Consistent with the high persistent positivity of high aPL titers and low persistent positivity of the low titers, we observed that samples that were negative at the first determination but close to cutoff tended to be positive at a retest more frequently than negative samples that had lower titers. Negative aCL and aB2GP1 samples >10 CU were positive at a retest 7.1% to 14.1% of the time, while negative samples >15 CU were positive at a retest 14.0% to 23.1% of the time. A similar trend was observed for aPS/PT (**Supplementary Table 4**).

### aPL platform comparison

We compared the QUANTA Flash and ELiA platforms using 73 previously frozen serum or plasma samples and found good correlation between the platforms. Correlation coefficients ranged from 0.6039 for aCL IgG to 0.9091 for aB2GP1 IgA (**Table 2**). Overall, the two platforms were more concordant at high aPL titers than low titers (data not shown).

**Table 2:** Correlation between assay platforms.

Correlation coefficient		
aCL	IgG	0.6039
	IgM	0.6963
	IgA	0.7394
aB2GP1	IgG	0.8213
	IgM	0.7962
	IgA	0.9091

Pearson correlation coefficients between the isotypes (IgG, IgM, and IgA) of anti-cardiolipin antibodies (aCL) and anti-beta2 glycoprotein-1 antibodies (aB2GP1) measured by chemiluminescence (QUANTA Flash; Werfen) and ELiA fluorescence enzyme immunoassay (Phadia; ThermoFisher Scientific) (N=73).

### Correlation with PC4d

We evaluated the correlation between PC4d and aPL in the cohort of patients for whom PC4d and aPL were measured. Correlation between PC4d and IgG was moderate even when only samples positive for a certain aPL were analyzed (Person correlation coefficients: 0.2649, 0.1917, and 0.2622 for the IgG of aCL, aB2GP21, and aPS/PT, respectively). Correlation was lower for IgM and was poor for the IgA isotypes (**Table 3**).

**Table 3:** Correlation between aPL and PC4d.

		All samples N	All samples Correlation coeff.	aPL positive samples N	aPL positive samples Correlation coeff.
aCL	IgG	6,912	0.1764	647	0.2649
	IgM	6,911	0.1049	330	0.1600
	IgA	6,403	0.1483	198	0.0192
aB2GP1	IgG	6,912	0.1841	435	0.1917
	IgM	6,912	0.0999	201	0.1009
	IgA	6,402	0.1198	171	0.0409
aPS/PT	IgG	6,400	0.2368	316	0.2622
	IgM	6,400	0.2448	964	0.1902

Pearson correlation coefficients between PC4d and the isotypes (IgG, IgM, and IgA) of anti-cardiolipin antibodies (aCL) and anti-beta2 glycoprotein-1 antibodies (aB2GP1) measured by chemiluminescence (QUANTA Flash; Werfen) or anti-phosphatidylserine/prothrombin complex antibodies (aPS/PT) measured by ELISA (QUANTA Lite; Werfen). All samples N and All samples Correlation coeff. refer to the analysis that included all samples, regardless of whether they were positive or negative for a certain aPL. aPL positive samples N and aPL positive samples Correlation coeff. refer to the analysis that included the subset of samples positive for the respective aPL.

## Discussion

To our knowledge, this is the first study that evaluated aPL persistent positivity in a large population of patients from the United States for whom aPL were tested for routine clinical assessment. In addition, we evaluated whether a marker of complement activation, PC4d, which is associated with thrombosis in SLE, can aid APS diagnosis.

Our large-scale analysis of positive aPL patients demonstrated that high titers of all isotypes of aCL and aB2GP1 are persistently positive in the vast majority of patients. In fact, when data were stratified by quartiles, over 80% of patients with titers in the two highest quartiles were positive at retest. Persistent positivity rate of the non-criteria aPS/PT was only slightly lower.

The interval of time between determinations in our dataset was variable and ranged from 0 to 252 weeks. This is not surprising, as our analysis was based on real word data. We hypothesized that high titers of aPL are persistently positive and, thus, that repeating aPL determinations after 12 weeks or longer is not necessary when titers are high. To test this hypothesis, we included all samples in the analysis, regardless of the length of time between determinations, although 92% to 95% of samples, depending on the particular analyte, were tested more than 12 weeks apart. Restricting the analysis to samples retested for aCL IgG and aB2GP1 IgG more than 7 days, or 30 days, or 12 weeks apart did not substantially change the results of the quartile analysis.

When evaluating predictors for aPL positivity at retest, the strongest predictor was the initial quantitative positive value of the same analyte, reinforcing the concept that aPL at high titers tend not to fluctuate over time. Our results are in agreement with what previously shown in a smaller patient cohort where approximately 80% of patients with clinically meaningful aPL profiles remained aPL positive at follow-up (15).

Also in agreement with previous data (25), double positive samples (positive for aCL and aB2GP1 IgG) were positive at retest with higher frequency than samples positive only for aCL or aB2GP1 IgG.

The finding that high aPL titers are often persistently positive suggests that a retest may not be necessary to confirm positivity. The clinical and treatment implications of these data are earlier diagnosis, risk assessment, and initiation of anticoagulation therapy in patients with APS at the time of the initial screening tests without the need for repeat testing at 12 weeks, as required by the APS classification criteria (5).

Low aPL titers may not be clinically significant for risk of thrombosis and pregnancy morbidity, and patients with APS or SLE with low aPL titers may be at lower risk (4, 15). Our data show that positivity rate at retest for positive samples in the lowest two quartiles was less than 50%. In addition, negative samples close to cutoff gave positive results at retest up to approximately 20% of the times. Taken together, these data indicate that aPL retest may be necessary for low positive and high negative titers when an autoimmune disease is suspected.

Several commercially available assays exist for the measurement of aCL and aB2GP1. Assays are not homogenously standardized and agreement between assays is fair to moderate (17, 26, 27). As different platforms may yield different results, we evaluated the correlation between the QUANTA Flash and the EliA platforms for aCL and aB2GP1. Consistent with previous data (17, 26), agreement between these platforms was good (correlation coefficients: 0.6039 to 0.9091). The high correlation between two methodologies suggests that our findings on aPL persistent positivity - or lack thereof - over time are independent of assay platform.

Both PC4d and aPL associate with thrombotic events in SLE (14, 23) and a modest correlation between PC4d and aPL in patients with SLE (22, 28) and in patients with APS or aPL positive but without APS (28) has been reported. We evaluated the correlation between PC4d and aPL in our large cohort of patients for whom PC4d and aPL were measured. Consistent with literature data (28), PC4d and aPL were at best moderately correlated even when only positive aPL samples were analyzed. These data suggest that PC4d and aPL have additive value to evaluate association with thrombosis in autoimmune diseases.

A limitation of our study is the inability to correlate aPL or PC4d positivity rate with clinical events as samples were analyzed as part of routine testing in the clinical laboratory and data on thrombotic events and obstetric morbidity were not available. However, association of aPL and PC4d with cardiovascular events in APS and SLE is well documented (2, 4, 12, 14, 23). In addition, data on LAC and aB2GP1 domain 1 antibodies were not available, and aPL persistent positivity could not be tested on the ELiA (Phadia) platform because, at the time of data analysis, a relatively small number of samples had been tested on this platform.

In conclusion, our analysis of a large cohort of patients demonstrates that high titers aPL are persistently positive. This observation has the potential to impact clinical practice, allowing an earlier diagnosis and risk assessment at the time of the initial screening. Conversely, a retest is necessary for low titers. The high correlation between two methodologies suggests that these findings are independent of assay platform. Finally, the low to moderate correlation between PC4d and aPL, combined with findings in previous studies (14, 22, 23, 28), might suggest a potential additive value to evaluate association with thrombosis in autoimmune diseases.

## Data availability statement

The raw data supporting the conclusions of this article will be made available by the corresponding author upon reasonable request.

## Ethics statement

Ethical review and approval was not required for the study on human participants in accordance with the local legislation and institutional requirements. Written informed consent from the participants’ legal guardian/next of kin was not required to participate in this study in accordance with the national legislation and the institutional requirements.

## Author contributions

RA wrote the manuscript and contributed to sample testing and data analysis. SS conceived the study. RB analyzed the data. TO contributed to data analysis. AK contributed to sample testing and data analysis. All authors contributed to the article and approved the submitted version.

## Acknowledgments

The authors wish to thank Joanne M. Yancon, Armida Sace, and Exagen’s laboratory personnel for technical assistance. We also thank Karina Baggiani and Debra Zack for the management and supervision of the clinical laboratory.

## Conflict of interest

RB, TO, AK, and RA are current or former employees of Exagen Inc. and/or stockholders of Exagen Inc.

The remaining author declare that the research was conducted in the absence of any commercial or financial relationships that could be construed as a potential conflict of interest.

## Publisher’s note

All claims expressed in this article are solely those of the authors and do not necessarily represent those of their affiliated organizations, or those of the publisher, the editors and the reviewers. Any product that may be evaluated in this article, or claim that may be made by its manufacturer, is not guaranteed or endorsed by the publisher.
