# Supplementary material for: Antiphospholipid antibodies are persistently positive at high titers. Additive value of platelet-bound C4d
Source: Front Immunol. 2022 Aug 10;13:949919. doi: 10.3389/fimmu.2022.949919 (PMC9399821; doi:10.3389/fimmu.2022.949919)
Supplement: Supplementary file 1 [file DataSheet_1.pdf]

SUPPLEMENTARY MATERIAL

ANTIPHOSPHOLIPID ANTIBODIES ARE PERSISTENTLY POSITIVE AT HIGH TITERS. ADDITIVE VALUE OF PLATELET-BOUND C4d

Supplementary Table 1. Time interval between determinations.

		Time between determinations (weeks)		
		Min.	Max.	Median (IQR)
aCL	IgG	0.00	250.00	56.14 (25.29-98.46)
	IgM	0.42	248.57	56.29 (26.29-104.00)
	IgA	1.14	248.71	57.14 (25.29-106.14)
aB2 GP1	IgG	0.00	248.71	54.07 (24.00-95.14)
	IgM	0.14	248.57	56.07 (25.68-102.00)
	IgA	1.71	248.71	55.07 (25.07-102.64)
aPS /PT	IgG	0.00	248.71	61.00 (30.71-106.71)
	IgM	0.00	251.86	58.00 (28.86-105.86)

Samples positive for the specified isotype (IgG, IgM, and IgA) of anti-cardiolipin antibodies (aCL), anti-beta2 glycoprotein-1 antibodies (aB2GP1), or anti-phosphatidylserine/prothrombin complex antibodies (aPS/PT) and for which a second determination was available were analyzed. Data is the time in weeks between the first positive measurement and the subsequent measurement. Data includes minimum (Min.), maximum (Max.), and median with interquartile range (IQR).

Supplementary Table 2. Positivity rate of aCL and aB2GP1 IgG at retest when samples tested at least 12 weeks, 30 days, or 7 days apart were included in the analysis.

	Interval between tests at least	Quartile	Percent
aCL IgG	12 weeks	1	41.9%
		2	68.2%
		3	84.3%
		4	95.5%
	30 days	1	43.1%
		2	68.4%
		3	84.8%
		4	95.8%
	7 days	1	43.5%
		2	68.7%
		3	84.8%
		4	95.7%
aB2GP1 IgG	12 weeks	1	38.1%
		2	68.8%
		3	88.5%
		4	98.2%
	30 days	1	38.9%
		2	67.8%
		3	88.8%
		4	98.0%
	7 days	1	39.5%
		2	68.1%
		3	88.7%
		4	98.1%

Samples positive for the IgG of anti-cardiolipin antibodies (aCL) and anti-beta2 glycoprotein-1 antibodies (aB2GP1) and for which a second determination occurred at least 12 weeks, 30 days, or 7 days apart were divided in quartiles based on their titers. *Percent* indicates the percent of positive samples that gave positive results at the first subsequent test in each quartile. aCL and aB2GP1 were measured by chemiluminescence (QUANTA Flash; Werfen) and were considered positive if > 20 chemiluminescent units (CU).

Supplementary Table 3. IgG persistent positivity in single or double positive samples.

Initial Positivity	Retest positivity definition	Positive samples at retest / total samples	Percent
aCL and aB2GP1 IgG positive	Either aCL or aB2GP1 positive at retest	925/980	94.4%
aCL IgG positive only	aCL positive at retest	1341/1984	67.6%
aB2GP1 IgG positive only	aB2GP1 positive at retest	421/686	61.4%
aCL IgG positive only	Either aCL or aB2GP1 positive at retest	1365/1984	68.8%
aB2GP1 IgG positive only	Either aCL or aB2GP1 positive at retest	431/686	62.8%

Samples positive for the IgG of anti-cardiolipin antibodies (aCL) and/or anti-beta2 glycoprotein-1 antibodies (aB2GP1) and for which a second determination was available were analyzed. *Percent* indicates the percent of positive samples that gave positive results at the first subsequent test. aCL and aB2GP1 were measured by chemiluminescence (QUANTA Flash; Werfen) and were considered positive if > 20 chemiluminescent units (CU).

Supplementary Table 4

		V0 > 10 units (N)	V0 > 10 units & V1 Positive (N)	Positivity rate (V0 > 10 units)	V0 > 15 units (N)	V0 > 15 units & V1 Positive (N)	Positivity rate (V0 > 15 units)
aCL	IgG	3,692	482	13.1%	1,336	309	23.1%
	IgM	1,646	160	9.7%	566	109	19.3%
	IgA	679	48	7.1%	229	32	14.0%
aB2GP1	IgG	1268	179	14.1%	373	86	23.1%
	IgM	718	75	10.4%	264	51	19.3%
	IgA	313	41	13.1%	109	25	22.9%
aPS/PT	IgG	4,540	185	4.1%	2,124	156	7.3%
	IgM	8,805	657	7.5%	5,221	586	11.2%

Analysis included samples negative for the specified isotype (IgG, IgM, and IgA) of anti-cardiolipin antibodies (aCL), anti-beta2 glycoprotein-1 antibodies (aB2GP1), or anti-phosphatidylserine/prothrombin complex antibodies (aPS/PT). Only samples for which a second determination was available were included in the analysis. *N* indicates the number of samples > 10 or > 15 units (chemiluminescent units [CU] for aCL aB2GP1 or units [U] for aPS/PT) at the first determination. *V0 > 10 units & V1 Positive (N)* and *V0 > 15 units & V1 Positive (N)* indicate the number of samples that were > 10 or > 15 units, respectively, at the first determination and that were positive at the second. *Positivity rate (V0 > 10 units)* and *Positivity rate (V0 > 15 units)* are the corresponding percentages. All aCL and aB2GP1 isotypes were measured by chemiluminescence (QUANTA Flash; Werfen) and were considered positive if > 20 CU. aPS/PT IgG and IgM were measured by ELISA (QUANTA Lite; Werfen) and were considered positive if > 30 U.
